# Identification of miRNA-mRNA network and immune-related gene signatures in IgA nephropathy by integrated bioinformatics analysis

**DOI:** 10.1186/s12882-021-02606-5

**Published:** 2021-11-25

**Authors:** Shi-Yao Wei, Shuang Guo, Bei Feng, Shang-Wei Ning, Xuan-Yi Du

**Affiliations:** 1 Department of Nephrology, Second Affiliated Hospital of Harbin Medical University, 246 Xuefu Road, Nangang District, Harbin, Heilongjiang Province 150086 People’s Republic of China; 2 College of Bioinformatics Science and Technology, Harbin Medical University, 157 Baojian Road, Nangang District, Harbin, 150081 Heilongjiang Province China; 3 Department of Nephrology, Fourth Affiliated Hospital of Harbin Medical University, Harbin, People’s Republic of China

**Keywords:** CIBERSORT, Hub genes, IgA nephropathy, Immune cells, Immunotherapy, microRNAs

## Abstract

**Background:**

IgA nephropathy (IgAN) is the most common form of primary glomerulonephritis worldwide, and its diagnosis depends mainly on renal biopsy. However, there is no specific treatment for IgAN. Moreover, its causes and underlying molecular events require further exploration.

**Methods:**

The expression profiles of GSE64306 and GSE93798 were downloaded from the Gene Expression Omnibus (GEO) database and used to identify the differential expression of miRNAs and genes, respectively. The StarBase and TransmiR databases were employed to predict target genes and transcription factors of the differentially expressed miRNAs (DE-miRNAs). Gene Ontology (GO) and Kyoto Encyclopedia of Genes and Genomes (KEGG) pathway analyses were conducted to predict biological functions. A comprehensive analysis of the miRNA-mRNA regulatory network was constructed, and protein–protein interaction (PPI) networks and hub genes were identified. CIBERSORT was used to examine the immune cells in IgAN, and correlation analyses were performed between the hub genes and infiltrating immune cells.

**Results:**

Four downregulated miRNAs and 16 upregulated miRNAs were identified. Forty-five and twelve target genes were identified for the upregulated and downregulated DE-miRNAs, respectively. CDKN1A, CDC23, EGR1, HIF1A, and TRIM28 were the hub genes with the highest degrees of connectivity. CIBERSORT revealed increases in the numbers of activated NK cells, M1 and M2 macrophages, CD4 naive T cells, and regulatory T cells in IgAN. Additionally, HIF1A, CDC23, TRIM28, and CDKN1A in IgAN patients were associated with immune cell infiltration.

**Conclusions:**

A potential miRNA-mRNA regulatory network contributing to IgAN onset and progression was successfully established. The results of the present study may facilitate the diagnosis and treatment of IgAN by targeting established miRNA-mRNA interaction networks. Infiltrating immune cells may play significant roles in IgAN pathogenesis. Future studies on these immune cells may help guide immunotherapy for IgAN patients.

**Supplementary Information:**

The online version contains supplementary material available at 10.1186/s12882-021-02606-5.

## Background

IgA nephropathy (IgAN) is the most common type of primary glomerulonephritis. However, there is no specific treatment for it. Nearly 40% of all patients with IgAN develop end-stage kidney disease within 20 years of disease onset [1]. Elevated proteinuria, hypertension, hypoproteinemia, hyperuricemia, and impaired renal function are independent predictors of a poor prognosis for IgAN [2]. The hallmark of renal biopsy is diffuse mesangial IgA deposition in the glomeruli. The most common manifestation is diffuse or segmental mesangial proliferation [3]. Effective treatments for IgAN are currently limited. A recent study showed that most IgAN patients have low circulation levels of galactose-deficient IgA1 (Gd-IgA1) [4]. New IgAN biomarkers that can identify individuals with the disease even before a renal biopsy is performed are required to assess and administer appropriate and timely treatment.

MicroRNAs (miRNAs) are small endogenous noncoding RNA molecules containing ~ 22 nucleotides, and they are involved in IgAN [5]. They undergo base-pairing with complementary sequences within mRNA molecules. MiRNAs regulate RNA silencing and post-transcriptional regulation of gene expressions. Hence, they participate in various cellular processes such as proliferation, differentiation, migration, and apoptosis. Several reports have demonstrated that the miRNA levels in peripheral blood monocytes (PBMCs) may be diagnostic indicators of IgAN. These include miR-148b, let-7b, miR-374b, and miR-155 [6–9]. Blood collection requires office visits, special tools, and trained professionals. In contrast, urine may be collected noninvasively and inexpensively and is an ideal screening specimen. Nevertheless, few studies have examined urinary miRNAs. Therefore, the aim of this study was to establish a reliable urine miRNA biomarker for IgAN diagnosis. To the best of our knowledge, there has been no systematic, comprehensive analysis of the miRNA-mRNA regulatory network based on clinical urine and kidney samples from patients with IgAN. The construction of a potential miRNA-mRNA regulatory network will help determine a comprehensive molecular mechanism through which miRNAs influence IgAN and may be used in the diagnosis of this disease.

In recent years, several studies have revealed that immune cell infiltration is crucial in the onset and progression of IgAN. The accumulation of B cells and T cells is implicated in IgAN. A study showed that the chemokine CXCL13 recruits CXCR5-positive B cells and contributes to the formation of renal lymphoid follicle-like structures comprising B cells and T cells [10]. Glomerular CD68^+^ cells of monocyte–macrophage lineage are putative markers of endocapillary hypercellularity [11]. Therefore, evaluating the infiltration of immune cells and differences in their components is important in detailing the molecular mechanism underlying IgAN and developing novel immunotherapeutic strategies. CIBERSORT is an analytical tool that evaluates immune cell infiltration using RNA-seq data, and it can identify the proportions of immune cells in various samples [12]. This tool has been widely used to analyze various diseases and conditions associated with immune cell infiltration such as systemic lupus erythematosus [13], kidney transplantation [14], and renal cell carcinoma [15]. However, no prior studies have used CIBERSORT to assess immune cell infiltration in IgAN.

## Methods

### Data collection and processing

The gene expression profile was downloaded from the Gene Expression Omnibus (GEO) database (http://www.ncbi.nlm.nih.gov/geo/). The GSE93798 dataset was from the study of Liu et al. [16], and the dataset was based on the platform of GPL22945 (Affymetrix Human Genome U133 Plus 2.0 Array). This dataset comprised kidney samples from 20 IgAN patients and 22 normal, healthy participants. The clinical data can be found in (Additional file 1: Table S1).

The miRNA expression profile under accession number GSE64306 was contributed by Wang et al. [17]. The dataset was downloaded from the GEO database according to the platform of the GPL19117 [miRNA-4] Affymetrix Multispecies miRNA-4 Array. MiRNA expression was analyzed in the urine sediments of 18 IgAN patients and six healthy individuals; the clinical information is shown in (Additional file 2: Table S2). Raw data from the GSE93798 and GSE64306 datasets were read using the “affy” package in R (version 3.5.3, R Core Team, Vienna, Austria). The RMA algorithm was used for background correction and data normalization. The comparison between the histopathological parameters of two dataset is shown in (Additional file 3: Table S3).

### Data processing and differential expression analysis

Microarray data were processed and analyzed in R. By comparing with the IgAN patients and normal individuals, we calculated the differentially expressed mRNA (DE-mRNA) from GSE93798 dataset and different expressed miRNA (DE-miRNAs) from GSE64306 dataset by “limma” package in R. In order to decreasing the false discovery rate, the *p* values were adjusted using the Benjamin and Hochberg method, the thresholds were adj.*p* < 0.05 and logFC > 1 (upregulated) or logFC < − 1 (downregulated). To visualize the DE-mRNA and DE-miRNA clusters, a heatmap of the expression patterns was plotted using the “pheatmap” package in R.

### Potential transcription factors (TFs) and target genes of the DE-miRNAs

The TransmiR database (http://cmbi.bjmu.edu.cn/transmir) was used to predict the upstream TFs of the DE-miRNAs. This tool is used in the study of TF-miRNA regulation and analyzes various processes, such as the evolution of interactions, expression patterns, and diseases associated with miRNAs [18]. The StarBase database (http://starbase.sys.edu.cn/) is a simple tool that provides query interfaces and graphical visualization pages and facilitates the analysis and exploration of miRNA-target interactions [19]. In this study, it was used to predict the downstream target genes of DE-miRNAs.

### Functional enrichment analysis

Gene Ontology (GO) is widely used to produce gene annotation terms [20]. The Kyoto Encyclopedia of Genes and Genomes (KEGG) is extensively applied in pathway enrichment analyses [21]. The selected DE-mRNAs were further evaluated for their corresponding biological meaning using GO annotations retrieved from the Gene Ontology Consortium, and the biological pathway information was mapped based on the KEGG database. The GO terms and KEGG pathways were analyzed with the “clusterProfiler” package and visualized with the “GOplot” package in R. *P* < 0.05 was considered statistically significant.

### Protein–Protein Interaction (PPI) network construction

The Search Tool for the Retrieval of Interacting Genes (STRING) (https://string-db.org/) was used to establish the PPI networks [22]. The STRING database was used to evaluate the correlations among DE-mRNAs. The overlapping DE-mRNAs were mapped in STRING to generate a network with functional interactions. Cytoscape software (version 3.6.1) was used to visualize the PPI network [23]. Then, the Molecular Complex Detection (MCODE) plugin was used to determine the most significant gene modules.

### Evaluation of infiltrated immune cells in IgAN

We adopted CIBERSORT method to determine the proportions of the 22 types of infiltrating immune cells in IgAN patients and normal controls [12]. This method mainly relies on a leukocyte gene signature matrix, called LM22, which contains 547 genes that differentiate 22 human hematopoietic cell phenotypes [12]. We analyzed the mRNA expression matrix using CIBERSORT R script acquired from the CIBERSORT website (https://cibersort.stanford.edu/), and employed the LM22 signature gene file and 1000 permutations as the reference. The Wilcoxon rank sum test was used to calculate the differences in the levels of immune cell infiltration between IgAN and normal samples (*P* < 0.05). Violin diagrams were plotted in the “vioplot” package in R to visualize the differences in immune cell infiltration between IgAN patients and normal controls. Pearson correlation analysis was implemented to obtain the related coefficient between the 22 immune cells. The “corrplot” package in R was used to plot a correlation heatmap to visualize the correlations of the 22 types of infiltrating immune cells between IgAN patients and normal controls.

### Correlation analysis between hub genes and infiltrating immune cells

The correlation between the hub genes and 22 immune cells was determined using Pearson’s correlation analysis and CIBERSORT. The Pearson analysis was performed in the “ggstatsplot” package in R (https://github.com/IndrajeetPatil/ggstatsplot). When *p* < 0.05, results were considered statistically significant, and the results were visualized with the “ggplot2” package in R.

### Data validation

In order to validate the reliability of the results of the current study, the gene expression profile GSE37460 were downloaded from the GEO database, based on the platform of GPL14663, (Affymetrix GeneChip Human Genome HG-U133A Custom CDF). This dataset comprised kidney samples from 27 IgAN patients and nine healthy participants. Different expressed genes (DEGs) between IgAN and normal individuals were identified and compared with the hub genes identified in GSE93798. In addition, CIBERSORT method was also used to analyze the mRNA expression matrix of dataset GSE37460, and the differences in the levels of immune cell infiltration between IgAN patients and normal samples were verified by Wilcoxon rank sum test (*P* < 0.05).

## Results

### DE-miRNA screening in IgAN patients and normal individuals

**Fig. 1:**
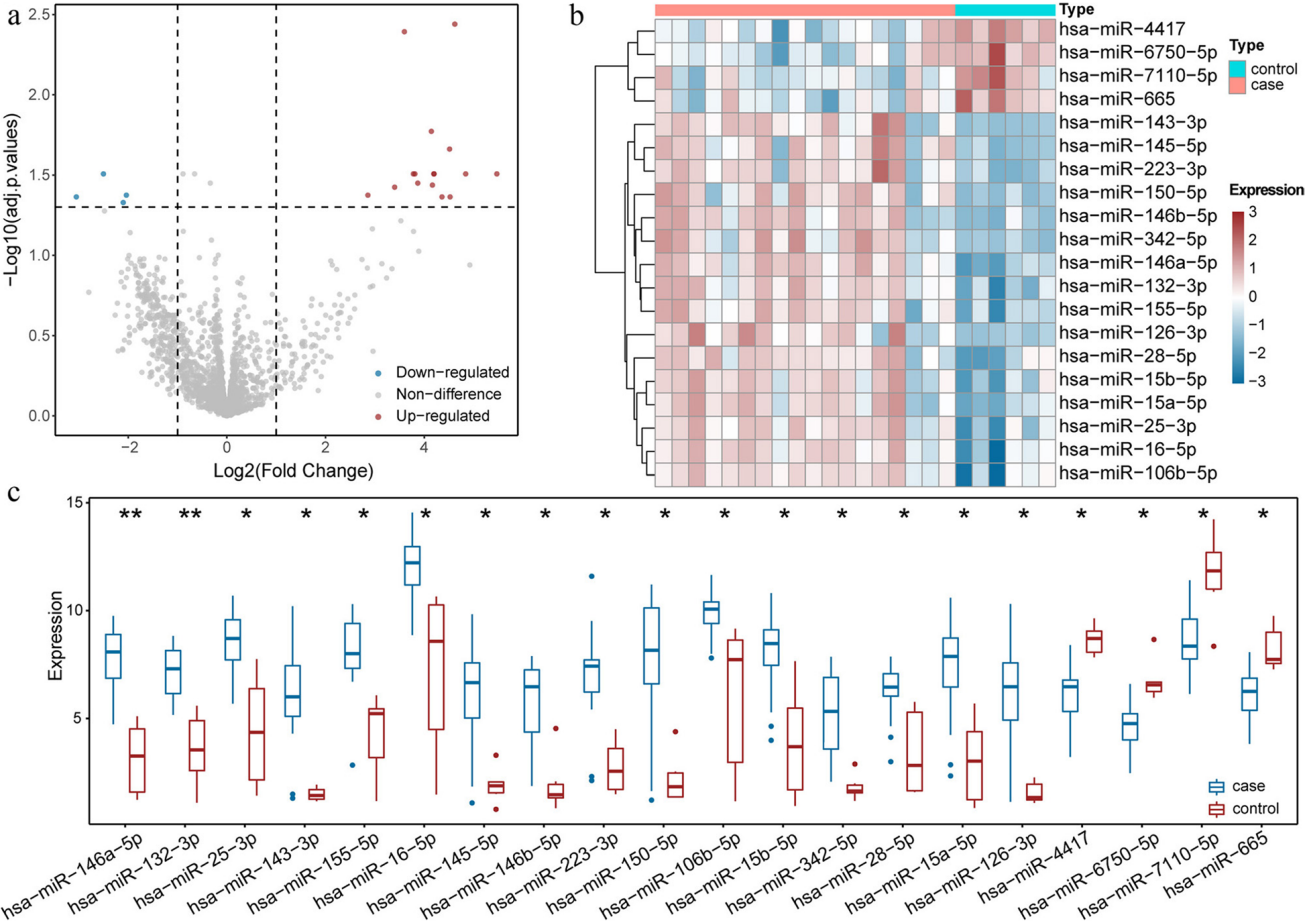
Identify DE-miRNAs in urine of IgAN patients. **a** Volcano plots showing the DE-miRNAs, the blue point indicate down-regulated and the red point indicate up-regulated. The dashed horizontal and vertical lines represent the cut-off criteria for significant regulation. **b** Heatmap of DE-miRNAs. Red indicates up-regulation and blue indicates down-regulation. In the above sample bar, the green represents adjacent normal samples and light red indicates IgAN kidney tissue. **c** Box plots show the level of DE-miRNAs. *P* < 0.05 was considered statistically significant, *P < 0.05, **P < 0.01,***P < 0.001

We first identified DE-miRNAs between IgAN patients and normal individuals in the GSE64306 dataset. There were 20 DE-miRNAs between IgAN patients and the normal controls. Of these, four DE-miRNAs were downregulated, whereas 16 DE-miRNAs were upregulated. The distribution of specific DE-miRNAs is illustrated with a volcano plot (Fig. 1a). We plotted a heatmap of the results of the cluster analysis of the DE-miRNA expression (Fig. 1b). The relative differences between groups in terms of miRNA expression are illustrated with box plots (Fig. 1c).

### DE-miRNA target gene and transcription factor predictions

**Fig. 2:**
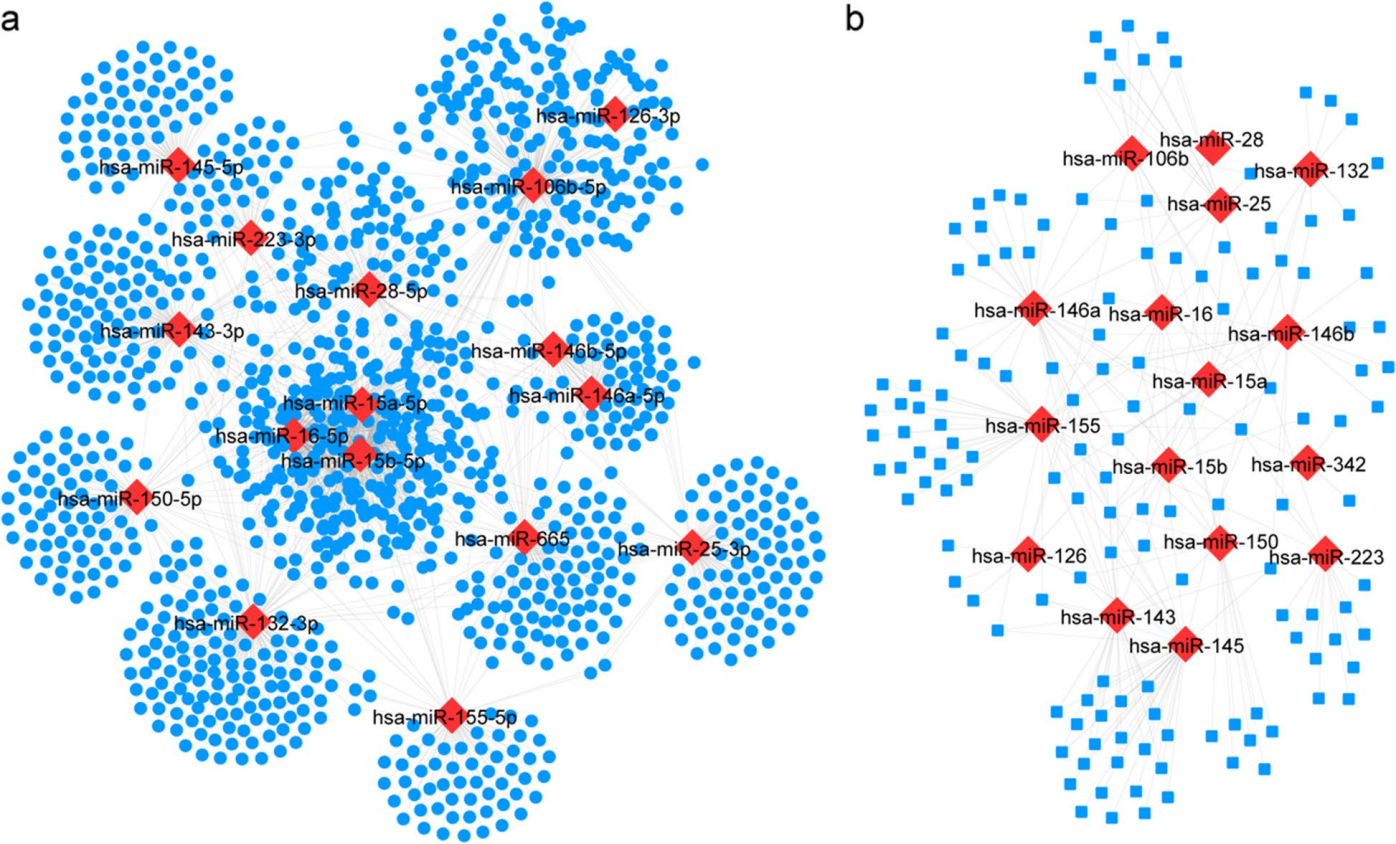
Construction of regulatory networks between DE-miRNAs and predicted target genes or transcription factors. **a** is the regulatory network of DE-miRNAs and predicted target genes. **b** is the regulatory network of DE-miRNAs and documented transcription factors. The red rhombus represent miRNAs, blue point represent predicted target genes, blue square represent transcription factors

We used the StarBase database to predict the downstream target genes of candidate DE-miRNAs and identify the potential miRNA-mRNA regulatory network in IgAN. We predicted 1221 target genes for the DE-miRNAs and have listed them in Additional file 4: Table S4. We constructed a regulatory network between the target genes and DE-miRNAs that consisted of 1237 nodes, 1938 edges, and 16 miRNAs (Fig. 2a). We used the TransmiR database to predict the upstream TFs of candidate DE-miRNAs (Additional file 5: Table S5). We also constructed a regulatory network between the TFs and DE-miRNAs that consisted of 156 nodes, 264 edges, 16 miRNAs, and 140 TFs (Fig. 2b).

### Identification of candidate target genes

**Fig. 3:**
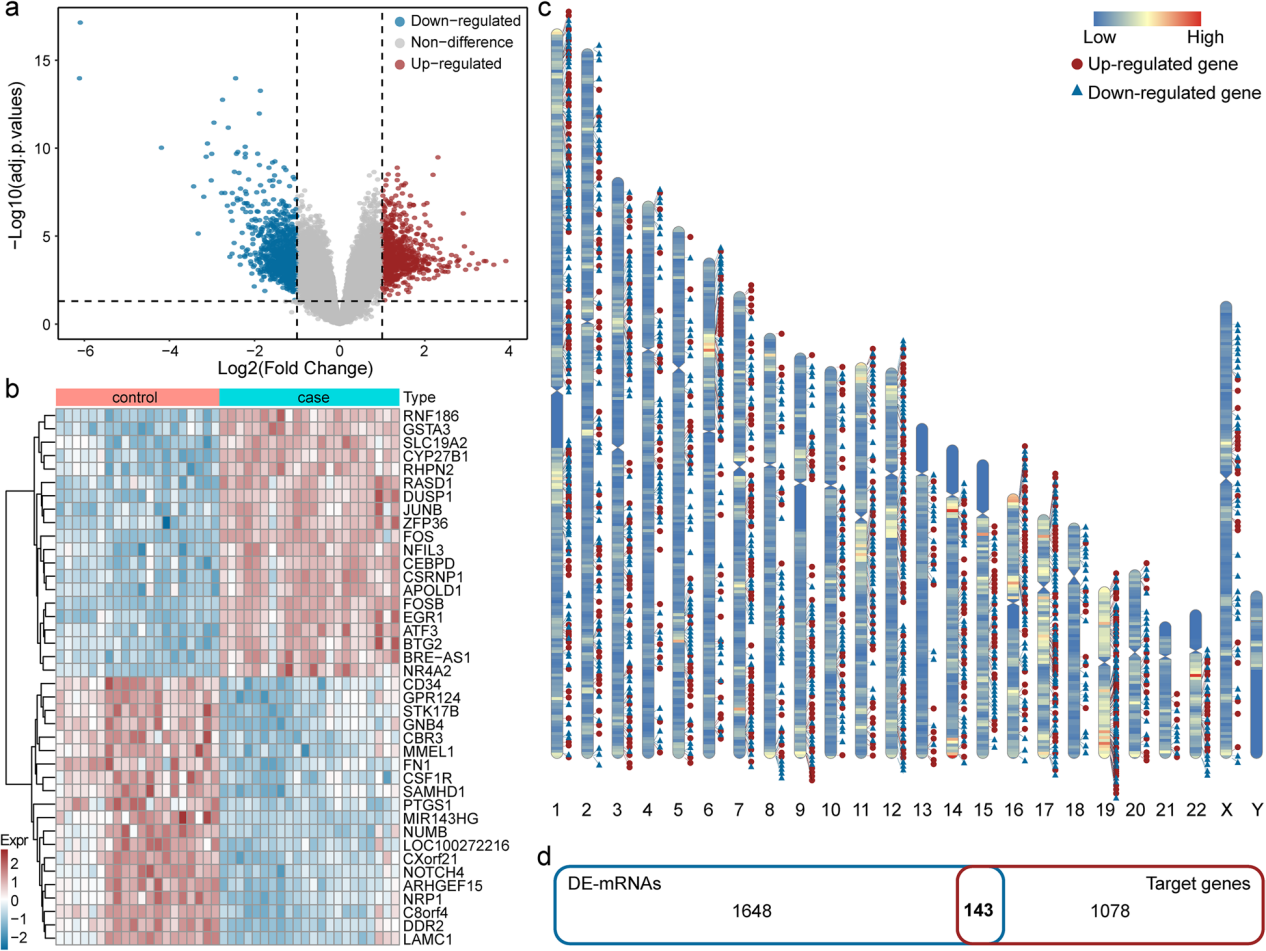
Identification of DEGs from tissue of IgAN patients. **a** Volcano plots showing the DEGs, the blue point indicate down-regulated and the red point indicate up-regulated. **b** Heatmap of DEGs. Red indicates up-regulation and blue indicates down-regulation. In the above sample bar, the green represents adjacent normal samples and light red indicates IgAN kidney tissue. **c** The distribution of differential expression genes binding sites across the whole human genome. **d** Intersection of differential expression genes in both sets

A differential expression analysis of mRNA (DE-mRNA) was performed on the kidney tissue (glomeruli) of IgAN patients and normal controls from the GSE93798 dataset in the GEO database. The results showed 957 downregulated and 834 upregulated genes between the IgAN patients and the normal controls. The distribution of specific DE-mRNAs is illustrated with a volcano plot (Fig. 3a). We selected the top 20 downregulated and upregulated genes and plotted a heatmap to show the results of the cluster analysis of DE-mRNA expression (Fig. 3b). To visualize the characteristics of the DEGs, we mapped the gene distribution across the entire genome at the chromosome level (Fig. 3c). We analyzed 1791 DE-mRNAs and 1221 of their target genes, and screened 143 candidate target genes common to both sets (Fig. 3d).

### Functional annotation and pathway enrichment analysis

**Fig. 4:**
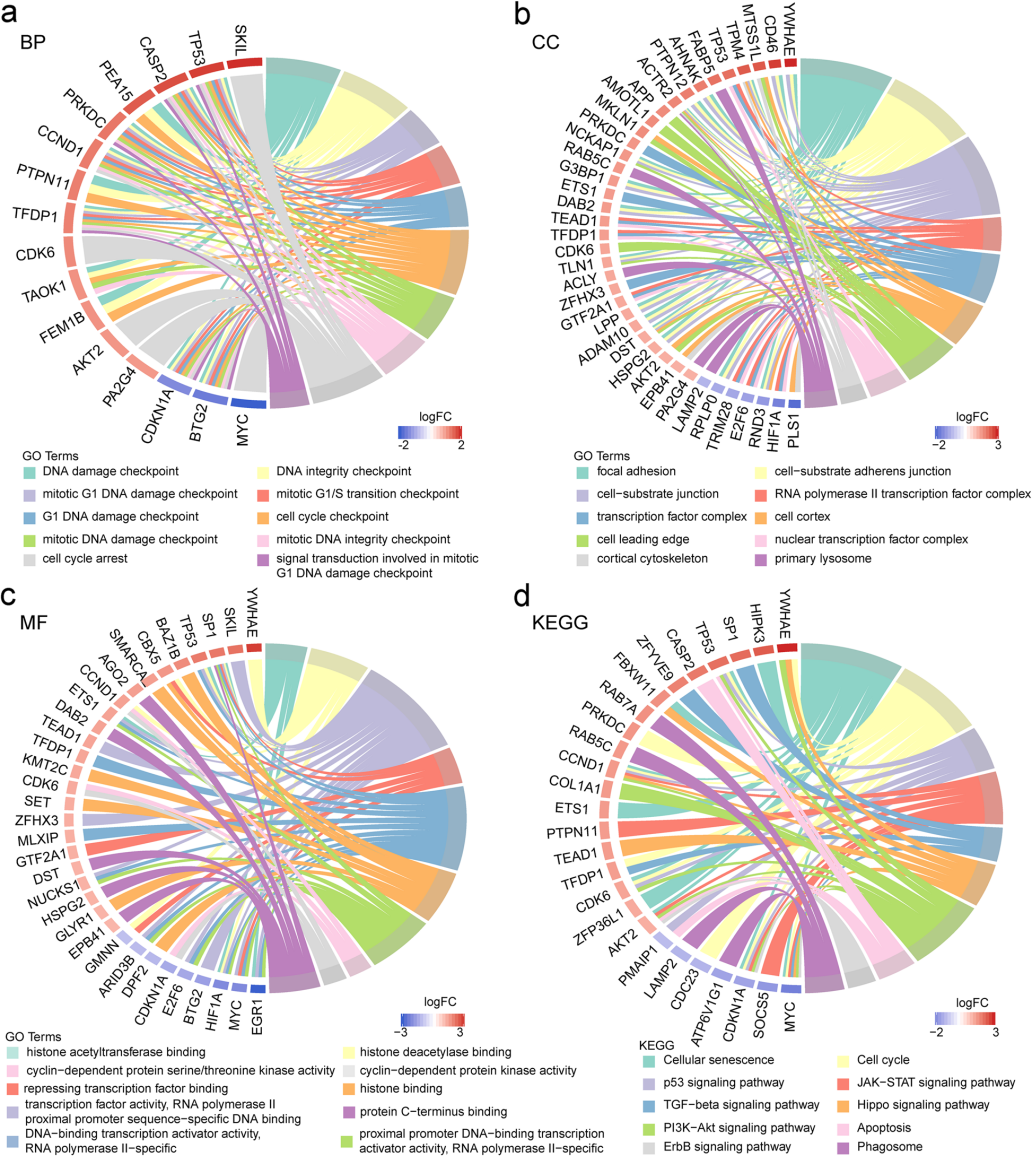
Enrichment analyses of candidate genes. **a**-**c** represents GO functional annotation. **a-c** showed the top 10 enriched BP items, CC items and MF items, respectively. **d** The enriched KEGG pathways of candidate target genes

To clarify the functions of the 143 target genes screened for IgAN, we uploaded them to the “clusterProfiler” package in R. The GO functional annotation included a biological process (BP), cellular component (CC), and molecular function (MF). The top 10 enriched GO items are shown in Fig. 4a–c. A GO BP analysis revealed that the candidate target genes were significantly enriched in the DNA damage, DNA integrity, mitotic G1 DNA damage, and mitotic G1/S transition checkpoints. The CC analysis revealed that the candidate target genes were enriched in focal adhesion, cell-substrate adherens and cell-substrate junctions, and the RNA polymerase II transcription factor complex. The MF analysis revealed that the candidate target genes were significantly enriched in histone acetyltransferase and histone deacetylase binding, transcription factor activity, and repressing transcription factor binding. These significantly enriched GO terms can help detail the roles of the screened target genes in IgAN onset and progression.

A KEGG pathway enrichment analysis was performed on the screened target genes (Fig. 4d). The candidate target genes were significantly enriched in cellular senescence, cell cycle, the p53, JAK-STAT, TGF-β, Hippo, PI3K-Akt, and ErbB signaling pathways, apoptosis, and phagosome.

### Construction of a candidate target gene-miRNA interaction network

**Fig. 5:**
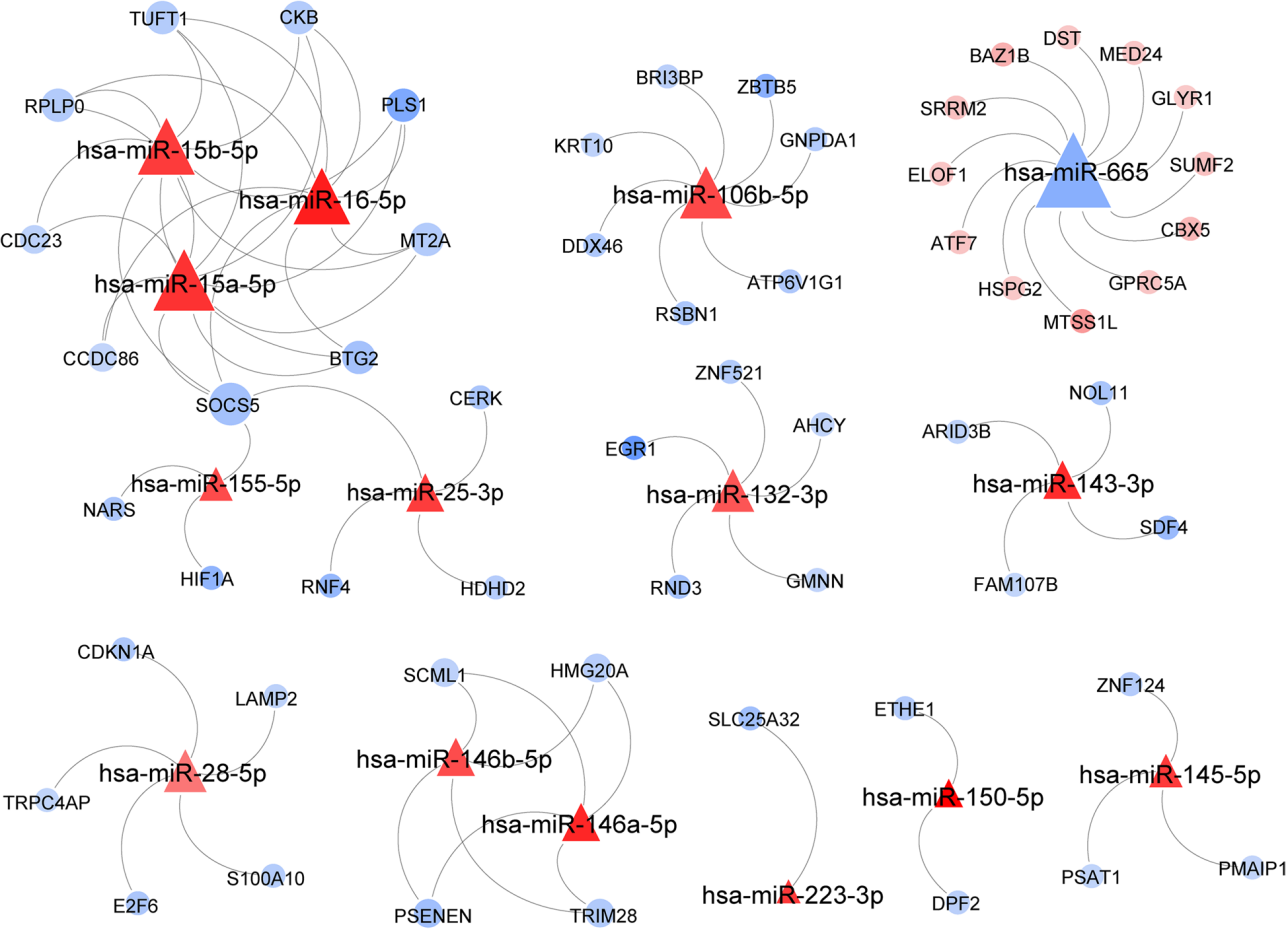
The candidate miRNA and target genes regulatory network in IgAN. Red triangles represent up-regulated, blue triangles mean down-regulated

An inverse relationship occurred between the miRNAs and target genes. Hence, we screened the intersection of the downregulated DEGs and predicted the target genes for upregulated miRNAs. We also screened the intersection of the upregulated DEGs and predicted target genes for downregulated miRNAs. Thus, we obtained 57 DEGs. We found 45 target genes for the upregulated DE-miRNAs and 12 target genes for the downregulated DE-miRNAs. Based on the predicted DE-mRNA-miRNA pairs, we constructed the candidate target gene-miRNA interaction network associated with IgAN onset and progression (Fig. 5).

### PPI network construction and screened hub genes

**Fig. 6:**
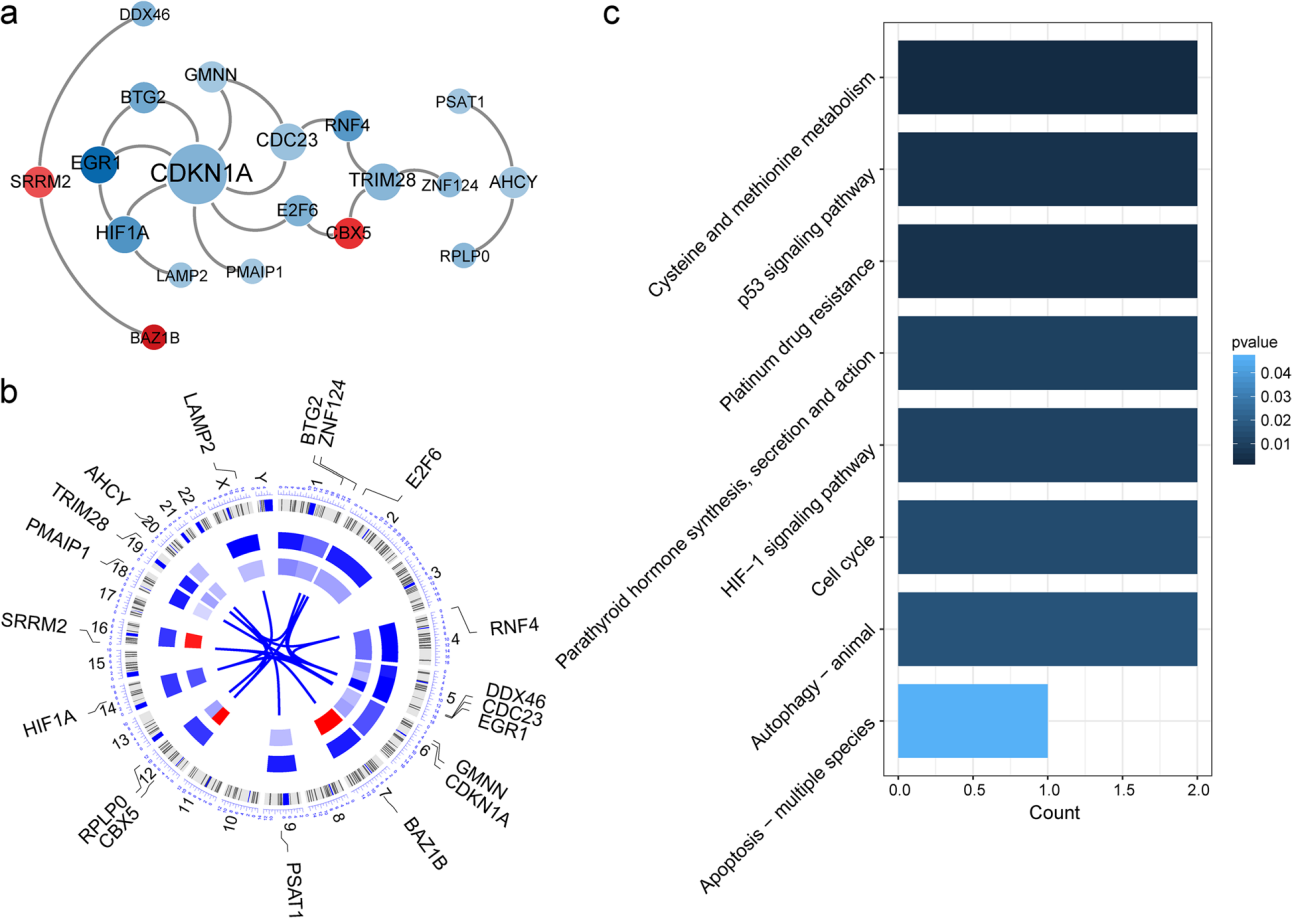
Construction of PPI network. **a** PPI network was constructed with hub genes. Red represents up-regulated, blue represent down-regulated. **b** Circos plot of the transcriptomic landscape of the PPI network genes. The outer ring shows chromosome ideograms with labelled chromosome identities. The scatter plot shows up- (Red) and down- (Blue) regulated genes. Gene fusions are shown as coloured arcs linking two genomic loci. **c** The enriched KEGG pathways for the hub genes

The 57 DEGs were entered into the STRING database for a PPI network analysis. Based on the string profile obtained from the STRING tool, the PPI network of the DE-mRNAs consisted of 19 nodes, 20 edges, and 19 genes (Fig. 6a). The top five hub genes possessing a high degree of connectivity in IgAN (TOP5) were as follows: CDKN1A (degree = 7), CDC23 (degree = 3), EGR1 (degree = 3), HIF1A (degree = 3), and TRIM28 (degree = 3). A co-expression map of the 19 genes across the entire genome is represented in a Cricos plot in Fig. 6b. A KEGG pathway enrichment analysis was conducted on the 19 candidate target genes of the DE-miRNAs (Fig. 6c). The enrichment pathways included cysteine and methionine metabolism, platinum drug resistance, the p53 signaling pathway, parathyroid hormone synthesis, secretion and action, the HIF-1 signaling pathway, the cell cycle, autophagy, and apoptosis.

### Immune cell infiltration results

**Fig. 7:**
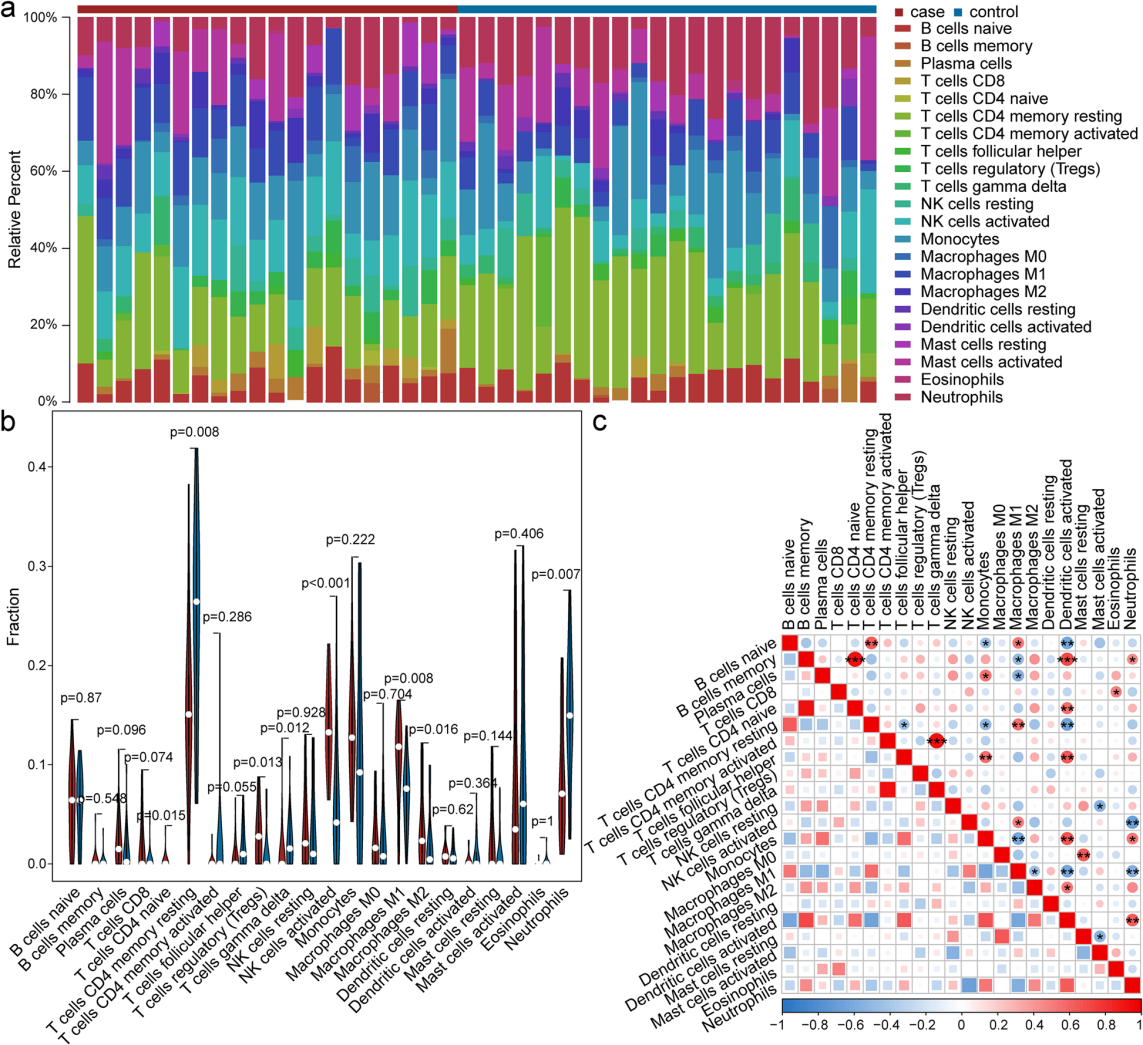
The landscape of immune infiltrationin in IgAN. **a** Relative proportion of immune infiltration in IgAN. **b**Violin plots visualizing significantly different immune cells in IgAN patients. **c** Correlation matrix of all 22 immune cells, the size of the colored squares represents the strength of the correlation; blue represents a negative correlation, red represents a positive correlation. *P* < 0.05 was considered statistically significant, *P < 0.05, **P < 0.01,***P < 0.001

CIBERSORT can be used to describe the cell compositions of complex tissues and their gene expression profiles, and can enumerate hematopoietic subsets in mixtures of RNA from fresh, frozen, and fixed tissues, including solid tumors [12]. The CIBERSORT method was used to estimate the differences in immune infiltration among the 22 immune cell types between IgAN patients and the normal controls. Figure 7a shows the proportions of immune cells in IgAN patients and the normal controls, respectively. Immune cell infiltration is illustrated with a violin plot. Relative to the normal control samples, activated NK cells, M1 and M2 macrophages, CD4 naïve T cells, and regulatory T cells (Tregs) were more infiltrated, while CD4 memory resting T cells, neutrophils, and γ-δ T cells were less infiltrated in the IgAN samples (Fig. 7b). A correlation heatmap of the 22 types of immune cells is shown in Fig. 7C. Several positive and negative relationships among 22 types of immune cells in the IgAN patients can be observed. B cell memory and T cells CD4 naive displayed the strongest positive correlation (correlation coefficient = 0.93), while NK cells activated and neutrophils showed the strongest negative correlation (correlation coefficient = − 0.67).

### Correlation between significant hub genes and infiltrating immune cells

**Fig. 8:**
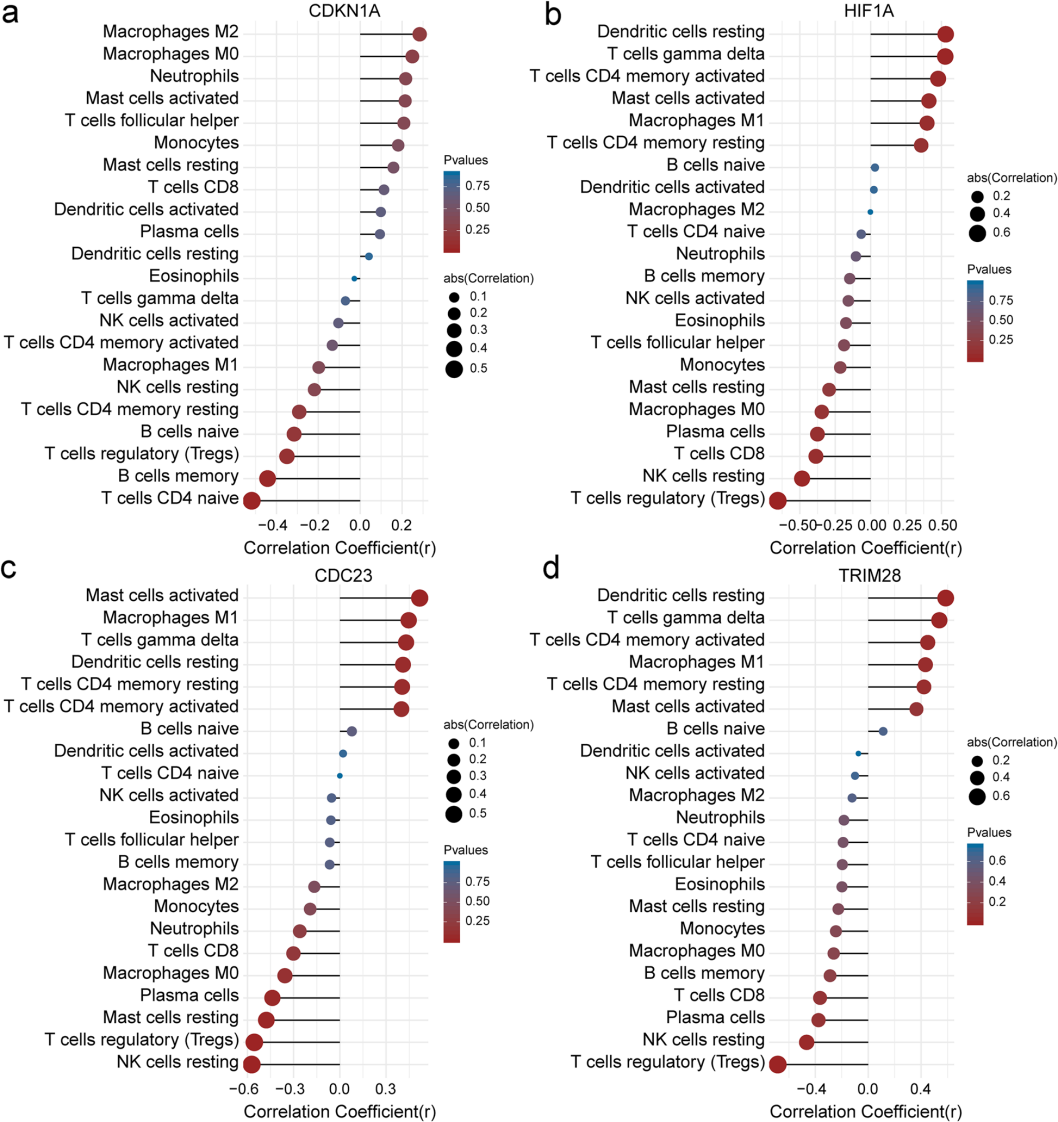
Correlation between CDKN1A, HIF1A, CDC23, TRIM28 and infiltrating immune cells. **a** Correlation between CDKN1A and infiltrating immune cells. **b** Correlation between HIF1A and infiltrating immune cells. **c** Correlation between CDC23 and infiltrating immune cells. **d** Correlation between TRIM28 and infiltrating immune cells. The size of the dots represents the strength of the correlation between genes and immune cells; the larger the dots, the stronger the correlation, and the smaller the dots, the weaker the correlation. The color of the dots represents the *p*-value, the redder the color, the lower the *p*-value, and more blue the color, the larger the *p*-value. *P* < 0.05 was considered statistically significant

A correlation analysis of the significant hub genes, such as CDKN1A, CDC23, EGR1, HIF1A, and TRIM28, and the infiltrating immune cells showed that CDC23, CDKN1A, HIF1A, and TRIM28 were significantly associated with infiltrating immune cells. CDKN1A was negatively correlated with CD4 naive T cells (*p* = 0.019; *r* = − 0.518) (Fig. 8a). HIF1A was positively correlated with activated CD4 memory T cells (*p* = 0.034; *r* = 0.476), γ-δ T cells (*p* = 0.017; *r* = 0.526), and resting dendritic cells (*p* = 0.016; *r* = 0.529), but was negatively correlated with Tregs (*p* = 0.002; *r* = − 0.655) and resting NK cells (*p* = 0.031; *r* = − 0.484) (Fig. 8b). CDC23 was positively correlated with M1 macrophages (*p* = 0.048; *r* = 0.447) and activated mast cells (*p* = 0.019; *r* = 0.518), but negatively correlated with Tregs (*p* = 0.011; *r* = − 0.555), resting NK cells (*p* = 0.008; *r* = − 0.572), and resting mast cells (*p* = 0.033; *r* = − 0.477) (Fig. 8c). TRIM28 was positively correlated with activated CD4 memory T cells (*p* = 0.046; *r* = 0.45), γ-δ T cells (*p* = 0.014; *r* = 0.538), and resting dendritic cells (*p* = 0.007; *r* = 0.586), but negatively correlated with Tregs (*p* = 0.001; *r* = − 0.684) and resting NK cells (*p* = 0.039; *r* = − 0.465) (Fig. 8d).

### Data validation

Data validation demonstrated that the hub genes obtained in GSE93798, such as CDKN1A, CDC23, EGR1, HIF1A, and TRIM28 were downregulated. And the downregulation of CDKN1A, EGR1 and TRIM28 were also observed in GSE37460 (Additional file 6: Fig. S1). In addition, the mRNA expression matrix of GSE37460 was analyzed by CIBERSORT to estimate the differences in immune infiltration among the 22 immune cell types between IgAN patients and normal individuals. Relative to the normal control samples, activated NK cells and M2 macrophages also demonstrated significant infiltration as based on the two datasets (Additional file 7: Fig. S2).

## Discussion

In current study, we built a miRNA-mRNA regulatory network based on aberrantly expressed urinary miRNAs and renal genes from IgAN patients. Several studies have demonstrated that miRNA-mRNA dysregulation contributes to kidney disease. Research on the involvement of miRNAs in the development of kidney disease is still relatively new but rapidly expanding. In recent years, it has become evident that miRNAs and the expression levels of their downstream target genes are potentially important in IgAN. They are expressed in both normal and diseased tissues and participate in kidney disease [24, 25]. The CIBERSORT tool has facilitated the analysis of immune cell infiltration patterns in various diseases. We used CIBERSORT to identify the roles of immune cell infiltration in IgAN and explore the relationships between the hub genes in the PPI network and infiltrating immune cells in IgAN.

We performed a differential expression analysis using miRNA and mRNA data from the GEO database. Four downregulated and 16 upregulated DE-miRNAs were identified in the urine sediments of IgAN patients and normal controls. Urinary miRNA detection is non-invasive, simple, inexpensive, and the miRNA levels are associated with various conditions, including renal allograft rejection, genitourinary tumors, and diseases native to the kidney [26, 27]. Intrarenal and urinary miRNA levels are closely correlated [28]. Several of the DE-miRNAs screened in this study were identical to those reported in previous studies. In IgAN patients, urinary miR-146a levels are inversely correlated with urinary interleukin (IL)-1β, IL-6, and tumor necrosis factor (TNF)-α expression, whereas urinary miR-155 levels are inversely correlated with urinary IL-1β and TNF-α expression [28]. However, a previous study found that urinary miR-146a and miR-155 levels were positively correlated with RANTES (regulated on activation, normal T cell expressed, and secreted) in IgAN patients [28]. Urinary miR-150 levels were confirmed using a NanoString miRNA assay and RT-qPCR, and were significantly upregulated in IgAN patients [29]. A previous study reported that in IgAN patients, miR-150-5p expression was associated with tubule-interstitial mononuclear infiltrates, especially in lymphoid nodules, and was largely located in areas of scarred cortexes and atrophic tubules; mesangial cells proliferation and parietal epithelial cells expressed modest miR-150-5p [30]. In addition, the study demonstrated that urinary miR-25-3p was significantly unregulated in IgAN patients. ROC curve analysis showed that the urinary sediment miR-25-3p had a high specificity (79.4%) and sensitivity (87.1%), which was suitable for diagnosing IgAN. By using CD235a immunomagnetic technology, urinary sediment miR-25-3p was found mainly derived from urinary erythrocytes [31]..

TFs bind miRNA promoters or enhancers and regulate their expression. We predicted the TFs that modulate DE-miRNAs and discovered that many of these genes were associated with the onset and progression of IgAN. Notch transmembrane receptor proteins (Notch1–4) are the main components of the Notch signaling pathway. A previous study reported that a cleaved Notch1 fragment expression in podocytes or glomeruli was correlated with albuminuria and glomerulosclerosis in various kidney disorders [32]. A recent investigation demonstrated that miR-146a plays an important regulatory role in podocytes by directly modulating the Notch1 and ErbB4 expressions [33]. Snai2 is a member of the snail TF family, and contains five consecutive *C*-terminal Zn fingers that facilitate binding to the E-box consensus CAGGTG motifs of the target genes. It also contains an evolutionarily conserved *N*-terminal SNAG domain that might epigenetically silence target genes [34]. A previous study confirmed that Snail is a key molecule perturbing the integrity of the slit diaphragm by transcriptionally repressing nephrin in a rat nephritis model induced by puromycin aminonucleoside (PAN) [35]. The roles of these predicted TFs in IgAN merit further investigation.

A previous study determined significant pathways by analyzing the DEGs of the GSE93798 dataset; most of the pathways were related to inflammation, cytokines, and growth factors such as the production of NO and reactive oxygen species in macrophages, interleukin (IL)-8 signaling, IL-6 signaling, and so on [16]. In the current study, we found some pathways that were differentially expressed from the results of Liu et al. [16]; the 143 candidate target genes for IgAN were discovered by integrating the DE-mRNAs and target genes of DE-miRNAs. The genes were enriched mainly in cellular senescence, the cell cycle, and the p53 and JAK-STAT signaling pathways. A few studies indicated that the kidney tissues of young patients with IgAN expressed senescence markers such as p16, p21, and senescence-associated-β-galactosidase (SA-β-gal). These markers were associated with interstitial tubule injury and poor renal function [36, 37]. A previous study indicated that the p53 signaling pathway is associated with the progression of IgAN and associated renal outcomes [38]. An earlier study reported that the dysregulation of cytokine levels is associated with the activation of the JAK-STAT signaling pathway, and the latter plays a role in IgAN pathogenesis and progression [39].

As there was an inverse relationship between the miRNAs and their target genes, we screened 57 candidate target genes, constructed a PPI network, and identified the top hub genes. GPRC5A is implicated in embryonic development, differentiation, tumorigenesis, and the maintenance of epithelial homeostasis [40]. A recent study reported that a GPRC5A knockout markedly reduced the proliferation and migration of progressive prostate cancer cells [41]. Another study showed that GPRC5A is highly expressed in podocytes but not in other types of kidney cells. GPRC5A regulates the EGFR and TGF-β signaling pathways in podocytes [42]. However, the roles of GPRC5A in IgAN development are unclear. Inflammation and hypoxia often coexist and regulate each other. Studies indicated that HIF may play a modulatory role in inflammation in kidney injury and repair [43]. HIF-1α is ubiquitously expressed in the organs of most cell types. HIF-1α was expressed in most renal epithelial cells in kidneys [43, 44], whereas HIF-2α was expressed tissue limited, and it has been detected in highly vascularized tissues and organs. HIF-2α is mainly expressed in renal interstitial fibroblast-like cells and endothelial cells [44, 45]. However, no study has reported on the roles of the HIF pathway in IgAN glomeruli. EGR-1 is a Zn-finger TF expressed in various eukaryotic cells. EGR-1 has been reported expression in many types of kidney cells; the expression pattern depends on where the primary site of injury is in the animal model [46]. In the model with 5/6 nephrectomy, EGR-1 was expressed in renal tubular epithelial cells [47], and in the model of mesangioproliferative glomerulonephritis, EGR-1 was expressed in the mesangial cells [48]. Nevertheless, the roles of EGR-1 in IgAN glomeruli are unknown. The target genes require validation, and the relationships between them and IgAN prognosis merit further investigation. We established a potential miRNA-mRNA regulatory network based on these findings. The expression patterns and effects of these miRNAs and the mRNAs associated with IgAN were previously confirmed. However, few of the miRNA-mRNA pairs that are listed in the network and contribute to IgAN pathogenesis have been investigated.

In the present study, we used CIBERSORT to elucidate the differential expression of immune cell infiltration patterns in IgAN. Increased infiltration of activated NK cells, M1 and M2 macrophages, CD4 naïve T cells, and Tregs and decreased infiltration of γ-δ T cells, resting CD4 memory T cells, and neutrophils may be associated with the onset and progression of IgAN. A recent study showed that IgAN patients presented with higher proportions of CD56^dim^CD16^+^ NK cells than normal controls. Hence, NK cells might be correlated with IgAN pathogenesis [49]. An earlier study showed a positive correlation between CD68^+^ macrophages and IgAN progression [50]. A recent study showed that M2a macrophages were positively correlated with proteinuria and serum creatinine. Moreover, the presence of M2a macrophages might aggravate IgAN progression [51]. Nonetheless, the roles of CD4 naïve, Tregs, γ-δ, and resting memory T cells in IgAN are unknown. The results of our study revealed the associations between the infiltration of 22 types of immune cells and IgAN progression. Activated NK cells are positively correlated with M1 macrophages and negatively correlated with neutrophils. M1 macrophages are negatively correlated with M2 macrophages, activated dendritic cells, and neutrophils. M2 macrophages are positively correlated with activated dendritic cells. Further research is required to clarify the mechanisms underlying these immune cell associations. Based on our correlation analysis between hub genes and immune cells, we speculate that HIF1A and TRIM28 are negatively correlated with Tregs and positively correlated with γ-δ T cells. We also propose that CDC23 is negatively correlated with Tregs, whereas CDKN1A is negatively correlated with CD4 naïve T cells. All these associations might contribute to the onset and progression of IgAN. Nevertheless, further research is required to detail the complex interactions between the aforementioned genes and immune cell infiltration.

The dataset of GSE37460 confirmed the hub genes and validated the infiltrated immune cells in this study; however, our study has certain limitations. First, the size of the sample set drawn from the GEO dataset was too small. Second, this study did not experimentally validate the analytical results or the direct relationships in the miRNA-mRNA pairs. Third, the CIBERSORT analysis was based on limited genetic data that may deviate from disease-induced disorders, heterotypic cellular interactions, and phenotypic plasticity; although the estimation bias of CIBERSORT was considerably lower than other approaches, some cell types were systematically overestimated or underestimated by CIBERSORT [12]. In a future study, experiments will be conducted to validate the results obtained in this study, and we will attempt to overcome the above limitations.

## Conclusions

In this study, we identified several miRNAs as urinary biomarkers of IgAN, performed an integrated analysis, and mapped a potential miRNA-mRNA regulatory network. The present study showed that miRNA-mRNA regulatory axes played important roles in the interaction networks related to IgAN pathogenesis. We will further verify the miRNA-mRNA network in future experiments and the findings of this study may assist in IgAN treatments by targeting established miRNA-mRNA interaction networks. We identified the core genes and used CIBERSORT to analyze immune cell infiltration in IgAN renal tissues. Infiltrating immune cells might play critical roles in IgAN pathogenesis. Further studies of these immune cells could potentially guide future immunotherapy regimes in patients with IgAN.

## Supplementary Information

**Additional file 1: Supplementary Table 1.** Clinical information of patients in GSE93798.

**Additional file 2: Supplementary Table 2.** Clinical information of patients in GSE64306.

**Additional file 3: Supplementary Table 3.** Comparison of the Oxford pathologic classification of patients in GSE64306 and GSE93798.

**Additional file 4: Supplemental Table S4.** Predicted Target genes of candidate DE-miRNAs.

**Additional file 5: Supplemental Table S5.** Predicted TFs of candidate DE-miRNAs.

**Additional file 6: Figure S1.** The expression level of 5 hub genes from the GSE37460 dataset.

**Additional file 7: Figure S2.** The validation of the infiltrating immune cells by GSE37460 dataset.

## Data Availability

The authors declare that the data supporting the findings of this study are available within the article and its supplementary information files. The datasets generated and analyzed during the current study are available in the GEO repository (GSE64306, GSE93798, and GSE37460). The gene expression profile was downloaded from the Gene Expression Omnibus (GEO) database (http://www.ncbi.nlm.nih.gov/geo/).

## Abbreviations

IgAN: IgA nephropathy
GEO: Gene Expression Omnibus
GO: Gene Ontology
KEGG: Kyoto Encyclopedia of Genes and Genomes
PPI: Protein-protein interaction
Gd-IgA1: Galactose-deficient IgA1
miRNAs: MicroRNAs
PBMCs: Peripheral blood monocytes
TF-miRNA: Transcription factors of miRNAs
DEGs: Differentially expressed genes
DE-mRNAs: Differentially expressed mRNA
DE-miRNAs: Differentially expressed miRNAs
STRING: Search Tool for the Retrieval of Interacting Genes
BP: Biological process
MF: Molecular function
CC: Cellular component
TNF: Tumor necrosis factor
PAN: Puromycin aminonucleoside
RT-QPCR: Real-time quantitative polymerase chain reaction
SA-β-gal: Senescence-associated-β-galactosidase
Notch1–4: Notch transmembrane receptor proteins
Tregs: Regulatory T cells
