# Supplementary material for: Identification of miRNA-mRNA network and immune-related gene signatures in IgA nephropathy by integrated bioinformatics analysis
Source: BMC Nephrol. 2021 Nov 25;22:392. doi: 10.1186/s12882-021-02606-5 (PMC8620631; doi:10.1186/s12882-021-02606-5)
Supplement: Supplementary file 1 — Additional file 1: Supplementary Table 1. Clinical information of patients in GSE93798. [file 12882_2021_2606_MOESM1_ESM.pdf]

Supplementary Table1：Clinical information of patients in GSE93798.

Sample	Age (year)	Sex	Urine Albumin to Creatinie Ratio	Creatinie (umol/L)	eGFR	Oxford classification			
						M	E	S	T
IgAN-1	54	female	229	132	39.4	0	0	1	1
IgAN-2	46	male	323	155	45.6	1	1	1	1
IgAN-3	45	female	155	132	40.5	1	0	1	1
IgAN-4	41	female	189	165	33.0	0	1	1	1
IgAN-5	49	male	37	124	58.4	0	1	0	1
IgAN-6	48	male	65	139	51.3	0	0	1	1
IgAN-7	58	male	70	107	65.6	0	0	1	0
IgAN-8	35	male	14	103	80.7	0	0	1	0
IgAN-9	58	male	61	117	67.9	0	0	1	2
IgAN-10	34	male	5	124	64.9	0	0	1	1
IgAN-11	53	male	74	96	77.4	0	1	0	0
IgAN-12	61	male	560*	84	86.0	0	0	1	0
IgAN-13	65	male	490*	93	74.0	0	0	1	1
IgAN-14	17	female	15	71	108.2	1	1	1	0
IgAN-15	30	male	75	91	97.1	0	0	1	0
IgAN-16	20	male	1.2	76	124.8	0	0	0	0
IgAN-17	23	female	0.6	68	109.3	0	0	0	0
IgAN-18	24	male	12	77	125.6	0	0	0	0
IgAN-19	23	female	13	66	113.4	0	1	1	0

*: Calculated from total urinary protein;

eGFR has been calculated using the CKD-EPI creatinine formula;

M: mesangial hypercellularity; E: endocapillary hypercellularity; S: segmental glomerulosclerosis;

T：tubular atrophy/interstitial
