# Supplementary material for: Identification of miRNA-mRNA network and immune-related gene signatures in IgA nephropathy by integrated bioinformatics analysis
Source: BMC Nephrol. 2021 Nov 25;22:392. doi: 10.1186/s12882-021-02606-5 (PMC8620631; doi:10.1186/s12882-021-02606-5)
Supplement: Supplementary file 2 — Additional file 2: Supplementary Table 2. Clinical information of patients in GSE64306. [file 12882_2021_2606_MOESM2_ESM.pdf]

**Supplementary Table 2：Clinical information of patients in GSE64306.**

| Sample | Age(year) | Sex | Lee’s grade |
|---|---|---|---|
| GSM1568359 | 25 | male | I－II |
| GSM1568360 | 20 | male | I－II |
| GSM1568361 | 25 | male | I－II |
| GSM1568362 | 26 | female | I－II |
| GSM1568363 | 35 | female | I－II |
| GSM1568364 | 31 | female | I－II |
| GSM1568365 | 27 | male | III |
| GSM1568366 | 28 | male | III |
| GSM1568367 | 26 | male | III |
| GSM1568368 | 25 | female | III |
| GSM1568369 | 47 | female | III |
| GSM1568370 | 41 | female | III |
| GSM1568371 | 45 | male | IV－V |
| GSM1568372 | 39 | male | IV－V |
| GSM1568373 | 25 | male | IV－V |
| GSM1568374 | 26 | female | IV－V |
| GSM1568375 | 51 | female | IV－V |
| GSM1568376 | 32 | female | IV－V |
