# Supplementary material for: Identification of miRNA-mRNA network and immune-related gene signatures in IgA nephropathy by integrated bioinformatics analysis
Source: BMC Nephrol. 2021 Nov 25;22:392. doi: 10.1186/s12882-021-02606-5 (PMC8620631; doi:10.1186/s12882-021-02606-5)
Supplement: Supplementary file 3 — Additional file 3: Supplementary Table 3. Comparison of the Oxford pathologic classification of patients in GSE64306 and GSE93798. [file 12882_2021_2606_MOESM3_ESM.pdf]

Supplementary Table3: Comparison of the Oxford pathologic classification of patients in GSE64306 and GSE93798

Oxford classification		GSE64306 (N=18)	GSE93798 (N=19)	*p*
**M**				0.151
	M0	11 (61.1%)	16 (84.2%)	
	M1	7 (38.9%)	3 (15.8%)	
**E**				0.090
	E0	17 (94.4%)	13 (68.4%)	
	E1	1 (5.6%)	6 (31.6%)	
**S**				1.000
	S0	5 (27.8%)	5 (26.3%)	
	S1	13 (72.2%)	14 (73.7%)	
**T**				0.089
	T0	10 (55.6%)	10 (52.6%)	
	T1	3 (16.7%)	8 (42.1%)	
	T2	5 (27.8%)	1 (5.3%)	

p values were **calculated using the Fisher's exact test;**

M: mesangial hypercellularity; E: endocapillary hypercellularity; S: segmental glomerulosclerosis; T：tubular atrophy/interstitial
