# Supplementary material for: Identification of miRNA-mRNA network and immune-related gene signatures in IgA nephropathy by integrated bioinformatics analysis
Source: BMC Nephrol. 2021 Nov 25;22:392. doi: 10.1186/s12882-021-02606-5 (PMC8620631; doi:10.1186/s12882-021-02606-5)
Supplement: Supplementary file 6 — Additional file 6: Figure S1. The expression level of 5 hub genes from the GSE37460 dataset. [file 12882_2021_2606_MOESM6_ESM.pdf]

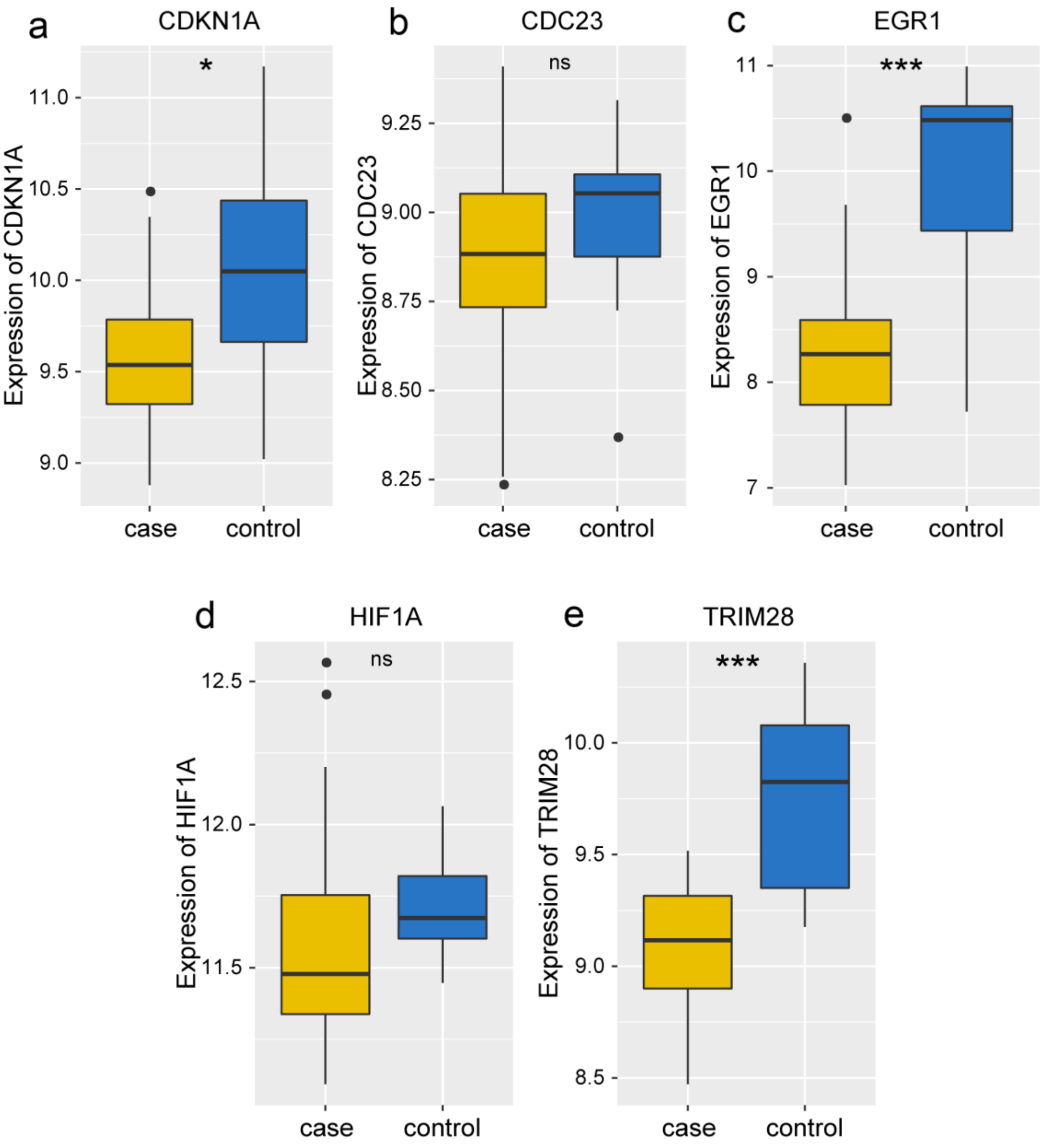

Figure S1. The expression levels of 5 hub genes from the GSE37460 dataset. P< 0.05 was considered statistically significant, *P < 0.05, **P < 0.01, ***P < 0.001.
